# Social contacts and loneliness affect the own age bias for emotional faces

**DOI:** 10.1038/s41598-022-20220-9

**Published:** 2022-09-27

**Authors:** Adriana Patrizia Gonzalez Pizzio, Alla Yankouskaya, Guido Alessandri, Sancho Loreto, Anna Pecchinenda

**Affiliations:** 1 Program in Behavioural Neuroscience, Department of Psychology, Sapienza” University of Rome, Rome, Italy; 2 Department of Psychology, Bournemouth University, Poole, UK; 3 Department of Psychology, Sapienza University of Rome, Rome, Italy; 4 Cognitive and Motor Rehabilitation and Neuroimaging Unit, IRCCS Santa Lucia, Rome, Italy

**Keywords:** Psychology, Human behaviour

## Abstract

Individuals are better at recognizing faces of their own age group (Own Age Bias) but it is unclear whether this bias occurs also for emotional faces and to what extent is affected by loneliness. Young individuals (N = 235) completed an age categorization task on faces of young and old individuals showing neutral, happy, and angry expressions. After a filler task, they categorized as seen or novel the original set of faces intermixed with a new set. Findings showed an Own Age Bias for novel young faces but no evidence that emotion eliminates it. Recognition accuracy was better for emotional faces, but the two factors did not interact. Importantly, low loneliness was linked to an Own Age Bias for novel happy faces. These findings are discussed in the context of current theoretical accounts of the Own Age Bias and of the effects of loneliness on attention and memory.

## Introduction

Human faces convey a wide spectrum of information, ranging from situational and transient information such as emotion and intention, to more stable information as identity, race, gender, or age^1,2^. This information is important for social interactions. Importantly, attention to faces can be affected by an individual’s characteristics^3,4^, which in turn can affect memory encoding and recall^5,6^. Accordingly, a well-known effect in face recognition is the so called own-group memory bias^7–9^ as people are better at remembering faces of their group.

Traditionally, there are two accounts for the Own-Group Bias. The social-cognitive evaluation account attributes the bias to differences in judgements for members of the own and other-group. More salient or positive evaluations of in-group members lead to preferential processing of own-group faces compared to out-group ones (for a meta-analysis see,^10^). For the perceptual expertise account, the bias is due to the greater contact and familiarity (experience) with individuals of the same group^11^. Most likely both accounts play a role in engendering the advantage for own-group faces (see the categorization-individuation model^8^). Albeit one the most studied features of Own-Group Bias is race, a memory bias for faces of one’s own age group (i.e., Own Age Bias) has also been observed.

The Own Age Bias refers to better recognition for individuals of similar age range as oneself^12^. In a meta-analysis, Rhodes and Anastasi^13^ concluded that the Own Age Bias is a robust effect for correct recognitions (i.e., higher hit-rates for same-age), false alarms (i.e., higher for other-age faces), and discriminability (i.e., higher for same-age faces), but not for response criterion (i.e., no clear tendency for conservative or liberal responses for facial age). Importantly, whereas the effect is strong for neutral faces, the pattern of results is quite complex for emotional faces. More specifically, some studies have observed an Own Age Bias (i.e., faster, and more accurate recognition) for emotional faces of young individuals^14–16^. In addition, Denkinger and Kinn^17^ observed the Own Age Bias for emotional and neutral faces in young as well as in old individuals. They asked participants (68 young and 19 older adults) first to rate the likeability of a set of 50 young and old faces with neutral, positive or negative expressions (emotion category not specified). After a filler task, participants performed a face recognition task (seen/not seen). Findings showed an Own Age Bias regardless of the facial expression, albeit the Own Age Bias was smaller for positive faces. In contrast, Ebner and Johnson^18^ failed to observe an Own Age Bias in young and old participants when using emotional and neutral faces. In their study, participants (32 young and 24 older adults) categorised old and young happy, angry, and neutral faces based on emotion, following which they completed a face recognition task (seen/not seen). In contrast, Cronin et al.^19^ have recently showed that emotional faces eliminate the Own Age Bias. They conducted 3 experiments using an intentional learning task at encoding (i.e., memorize faces) followed by a face recognition task (seen/not seen), separated by a 5-minute filler task. In experiment 1, neutral faces of young and old individuals were used, and findings showed an Own Age Bias (i.e., higher sensitivity to younger faces). In experiment 2 (72 participants) the same procedure was used with neutral and angry faces. Findings showed an Own Age Bias for neutral but not for angry faces. Finally, in experiment 3, neutral and sad faces (experiment 3a with 90 participants), or neutral and happy faces (experiment 3b with 90 participants) were used. Findings again showed an Own Age Bias, due to more accurate recognition for young neutral faces but not for young emotional faces.

Taken together these findings clearly show that an Own Age Bias occurs with neutral faces, but whether it also occurs with emotional faces is unclear. Importantly, the Own Age Bias should occur and even be enhanced for emotional faces when the need for social contacts and social affiliation with one’s peers is high. This assumption is based on recent work demonstrating that the need for social affiliation affects the neurobiology of emotional processing by activating affective and reward-related brain areas^20,21^. Moreover, it was suggested that the absence of positive social interaction may create a want, or ‘craving’, that directs behaviour to repair what is lacking by triggering emotional and motivation processes^22^. However, this has not been investigated yet in relation to the own-age bias in faces processing and the present study intends to fill this gap.

The research question stems from evidence that loneliness, that is the social pain engendered by perceived social isolation, motivates people toward signals of possible reconnection^23^. This is because lonely individuals show an acute need to re-establish social connections and prioritise affiliative signals^24,25^. However, lonely individuals are also sensitive to signals of social rejection and prioritise social threats^26–28^. Consequently, as happy and angry faces signal possible social affiliation and possible social threat respectively, they should be especially salient stimuli for lonely individuals. In addition, considering that the mechanisms underlying the Own Age Bias rely on expertise in social interactions with own-age individuals^12,29,30^ and/or on the salience of own-age individuals^8^, it is well possible that lonely individuals, who have fewer and less satisfying interactions with their peers^31^ show a reduced Own Age Bias.

## Method

All methods were carried out in accordance with relevant guidelines and regulations. All experimental protocols were approved by the Psychology Department Ethic Committee, Sapienza University of Rome. Informed consent was obtained from the study participants.

### Participants

Two-hundred and thirty-five university students aged between 18 and 30 years old (age M= 21.23 SD= 2.08; Males= 42, Females= 193) took part in the study. Sample size was a priori calculated using G*Power software^32^. We used the Own Age Bias effect size (d= .70, α = .05; β−1 = .95) reported by Cronin et al.^19^, which in turn is based on the Own Race Bias effect (η_p_^2^ = .11) reported by Gwinn et al.^33^. This established that with d = 0.70, α = 0.05, power = 0.951, 130 participants were sufficient to detect a moderate-large effect. In addition, calculations based on the effect size (t(63)= 4.14, *p*<.001, d_av_ = 0.64) reported in exp. 1 by Cronin et al.^19^, established that with d=.64, a=.05, power=.951, a sample size of 108 participants was sufficient. However, as the experiment was conducted online, we oversampled to 235 participants.

### Stimuli

Forty-eight identities (24 young adults: 12 males/12 females and 24 older adults: 12 males/12 females) were selected from the FACES database^34^. For each identity, neutral, angry, and happy expressions were selected for a total of 144 stimuli (72 young and 72 old). Young adult faces were between 22 and 33 years, older adult faces were between 61 and 80 years old. Images were in colour and 335 by 418 pixels in size. Based on available validation data^34^, the selected stimuli (see Supplementary Material) were balanced for distinctiveness, and expression accuracy. The 144 selected faces were divided in two equal sets of 72 faces (12 young faces and 12 older faces, all balanced for gender, with each of the 3 expressions), each set used for the encoding and test phases. As the FACES datasets has 2 versions of each face (version A and version B), each set of 72 faces was presented in both versions (if version A was presented at encoding, version B was presented at test phase as the “seen faces” together with the set of “new faces”). Which set of faces was used for encoding and test were counterbalanced across participants with two versions of the task (i.e., the 72 faces used for the encoding phase in one version of the experiment were used as “new faces” for the test phase in the other version of the experiment).

For male-faces, the selected young and old faces differed for age, t_(22)_= 26.471, *p*<.001 but were matched for expression accuracy, t_(22)_=1.417, *p*=.171 and distinctiveness, t_(22)_= 2.185, *p*=.74. For female-faces, the selected young and old faces did differ for age t_(22)_= 23.573, *p*<.001, but were matched for expression accuracy t_(22)_= 1.368, *p*=.185 and distinctiveness t_(22)_ =1.261 *p*=.221 (see Table 1).

**Table 1:** Mean ratings (SD) for young and older selected faces.

Rating	Male			Female		
	Young	Old	*p*	Young	Old	*p*
Age	28.30 (3.44)	68.36 (3.95)	< .001	26.54 (3.08)	68.34 (5.31)	< .001
Expression (accuracy)	92.42 (4.83)	88.92 (7.06)	.171	88.42 (10.67)	82.08 (8.27)	.185
Distinctiveness	34.98 (23.62)	35.76 (22.41)	.74	38.8 (23.73)	35.88 (23.02)	.221

### Questionnaires

The Italian version of the UCLA Loneliness Scale^35^ was used to assess loneliness. In addition, and for purposes not related to the hypotheses of the present study, two other questionnaires were used as a filler task. As in Cronin et al.^19^ participants were also presented with four questions aimed at assessing the frequency of social contacts with people of their own age group and with older people. Participants responded using an 8-point scale ranging from 1 (daily) to 8 (less than once a year). More specifically, the questions were: 1) How often do you have personal (i.e., face-to-face) contacts with young adults (approx. between 18 and 30 years of age)?; 2) How often do you have personal (i.e., face-to-face) contacts with older adults (approx. 65 years of age and older)?; 3) How often do you have other types of contact (e.g., phone, e-mail, letter) with young adults (approx. between 18 and 30 years of age)?; 4) How often do you have other types of contact (e.g., phone, e-mail, letter) with older adults (approx. 65 years of age and older)?

### Tasks

Face Task (encoding phase): There were 144 trials, consisting of 72 young (36 females and 36 males, each with neutral, happy, or angry expression) and 72 old faces (36 females and 36 males, each displaying neutral, happy, or angry expressions). Pictures were displayed one at a time for 1500ms. To make the encoding task less predictable, the intertrial interval randomly varied between 1000 and 1500ms. Participants were instructed to categorise each face based on age and responded by pressing “G” for GIOVANE (young) of “A” for ANZIANO (old)]. For each trial, the instruction “memorise the face” appeared below the face and remained onscreen the same as the face. Faces were displayed in a new random order for each participant.

Memory Task (test phase): There were 144 trials, consisting of 72 previously seen faces (12 young and 12 older faces) each showing either neutral, happy, or angry expressions intermixed with a new set of 72 faces (12 young and 12 old faces) each showing either neutral, happy, or angry expressions Faces were presented on screen one at a time for 10s and participants responded whether they had seen or not seen the face previously by pressing “V” for VISTA (seen) and “N” for NON VISTA (novel). Faces were displayed in a new random order for each participant.

As participants used their own keyboard to respond, to prevent labelling the keys, keys were chosen to be intuitive for each categorization task.

### Experimental design

The experimental design is a 2 (Face Age: young, old) × 3 (Face Emotion: neutral, happy, and angry) repeated measure design for each phase of the experiment.

### Procedure

The experiment was conducted online, using Testable (www.testable.org). The study was advertised using Moodle: students could read the study description on Moodle and sign up to complete the task online during the booked timeslots. On the day of the appointment, both participant and experimenter were connected via video-call (using Google Meet). After sending the link of the experiment, both participant and experimenter remained connected to the video-call via Google Meet.

Participants first read the description of the study and the experimenter answered eventual questions. Then, participants completed the informed consent, following which the questions of the UCLA Loneliness Scale^35^ and the 4 questions on social contact appeared onscreen. Participants completed the questionnaire before the Face Task to allow them to think about their social interactions. Upon completion of the questionnaire, the task instructions were presented onscreen. Participants were instructed to memorize a series of faces presented on screen as they would be asked to recognize them later. These intentional learning instructions are in line with other Own Age Bias studies^13^. To further facilitate encoding, participants were also asked to categorise each face as young or old as soon as the faces disappeared from the screen. Following the encoding phase, participants completed a filler task (i.e., questionnaires unrelated to loneliness) for approximately 5 mins. Next, the instructions for the face recognition task appeared onscreen. Participants were informed that they were going to see some faces, some of which they had already seen during the previous task, and some were novel. Their task was to indicate for each face whether they had already seen it or not seen it.

For both tasks, participants were asked to respond as quickly and accurately as they could. Finally, participants were thanked for the participation, were asked if they had questions, and were dismissed from the Google Meet video-call.

### Data analyses

#### Task performance measures

Two performance measures were used: response time (RTs) and accuracy for the Face Task (i.e., encoding phase) and the Memory Task (i.e., test phase). Data were analysed separately for each phase. Means RTs for correct responses were computed for each experimental condition. Accuracy, computed as corrected recognition (Pr), was calculated by subtracting the false alarm rate from the hit rate. For seen faces, false alarm rates represented the proportion of test trials in which the participant incorrectly responded ‘seen’ to a face that had not been seen before. Hit rates were computed as the proportion of test trials in which the participant correctly responded ‘seen’ to a face that was seen before. For novel faces, hit rates represented the proportion of novel faces that were correctly recognised as ‘novel’, whereas false alarm rates represented the proportion of previously seen faces that were recognised as ‘novel’.

#### Loneliness and social contacts

For the Italian adaptation of the UCLA^35^ a total score was computed after reverse scoring five items: higher scores indicate more loneliness. To assess whether number of social contacts can predict loneliness scores, we carried out a multiple regression analysis with number of social contacts with young and older people as independent variables and Loneliness scores as dependent variable. We used backward as the method of data entry and tested two models where one model included both independent variables, the second model contained only one independent variable. To test whether Own Age Biases in recognition of emotional faces could predict loneliness we used multiple regression analysis. We calculated the biases as difference in recognition scores between (i) Seen Angry Young faces and Seen Angry Old faces; (ii) Seen Happy Young faces and Seen Happy Old faces; (iii) Novel Happy Young faces and Novel Happy Old faces; (iv) Novel Angry Young and Novel Angry Old faces. These biases were entered as predictors and Loneliness scores as dependent variable.

#### Reliability analysis

Recent work demonstrated that high measurement error for response time and accuracy could be detrimental to the analysis and the inferences drawn from it^36,37^. Therefore, prior to data analysis, we assessed the reliability of our measurements by estimating the internal consistency of accuracy and response times for the test phase. We used a permutation-based split-half approach with 5000 random splits^38,39^. In the split-half method, the data for a measure is split into two halves. The Pearson correlation between these halves with subsequently applied the Spearman-Brown (prophecy) correction for the underestimations resulting from splitting the number of observations in half is then calculated as an estimate of the measure’s internal reliability.

#### Linear mixed modelling

To test the hypothesis that there is Own Age Bias for neutral but not for emotional faces, we applied a linear mixed modelling approach (LMM)^40,41^. The advantage of using this approach here is twofold. First, compared to a classical analysis of variance (ANOVA), LMM suffers less loss of statistical power if there are missing data^42^, and in the test phase there is a small proportion of missing RT data. Second, LMMs allow us to estimate fixed effects and their interaction and simultaneously, parameters of the variance and covariance components of random effects due to subjects^43^. The random effect of subjects, which is the subjects’ deviations from the grand mean accuracy, and RT and subjects’ deviations from the fixed-effect parameters is also of substantive theoretical interest here as it can provide future research with an important heuristic for identifying the sources of experimental effects. The contribution of the random effect of subjects was estimated using the Likelihood Ratio Test (LRT)^44^. The likelihood ratio statistic is equal to two times the difference of the log-likelihoods of two models, where one model includes a parameter of interest (fitted model), and the second model (null-model) does not contain the parameter of interest. Normality in the distribution of the residuals of final models was assessed using quantile-quantile plots.

Each LMM model in the Faces Task included three fixed terms (Age (Old, Young), Emotion (Angry, Happy, Neutral), interaction between Age and Emotion (Age*Emotion)) and a random effect of Subjects to account for idiosyncratic variation that is due to individual differences. To incorporate categorical effects from factors with discrete levels into the LMMs, we based our analyses on contrasts, which allow us to code factors as independent variables in linear regression models. We used a simple coding scheme to contrast levels of the Age factor ([Old-Young]) and Helmert coding scheme to contrast levels of the Emotion factor ([Neutral-(Angry, Happy)] and [Angry-Happy]). All LMM models were estimated using Jamovi version 2.2 (The jamovi project (2020). [Computer Software]. https://www.jamovi.org).

#### Face task (encoding phase)

To test the effects of Age of faces (Old, Young) and Emotion (Angry, Happy, Neutral) on categorisation performance, we modelled accuracy and response time using two separate LMM models. A fitted model included accuracy or response time as a dependent variable, three fixed effects (Age of faces, Emotion and interaction term Age*Emotion) and one random effect term (Subject).

#### Memory task (test phase)

First, to get an overall understanding of the performance and the central tendency of responses in the Face Task we used a bootstrapping procedure^45^. For each condition, we paired RT (x) and accuracy (y) for each participant. The data sets were then resampled with a replacement but kept the sample size as the number of participants. This procedure was repeated 2000 times, and each resampled set was plotted as a single data point. This procedure allowed us to visualise both the mean and variance of the data for each condition and their overlap indicating potential bias effects. The bootstrapping procedure was performed separately for seen and novel faces. Second, four separate LMMs were used to estimate the effects of Age of faces (Old, Young) and Emotion (Angry, Happy, Neutral) on accuracy and response time for seen and novel faces.

## Results

### Face task (encoding phase)

*Accuracy (Pr)* Overall, participants were accurate in categorising the age of faces (M_old_=0.97, SD=0.05; M_young_=0.98, SD=0.04). A LMM on Pr indicated that including a random effect of Subject benefited the model (LRT=190.01, df=1, *p* < .001). The results of fixed effects showed no effect of Age (F(1,1170) =1.94, *p*=.16) and Emotion (F(2,1170)=2.12, *p*=.1). The interaction between Age and Emotion was also non-significant F(2,1170) = 0.34, *p*=.71). A random effect of Subject explained 29% of the overall variation in accuracy responses.

*Response Times (RTs)* A LMM on RTs revealed no significant fixed effect of Age (F(1,1170)=0.21, *p*= .87, Emotion (F(2,170)=1.0, *p*=.34 and Age by Emotion interaction (F(2,1170)=0.56 *p*=.61). A contribution of a random effect of Subject was significant (LRT=1712.16, df=1, *p*<.001) and explained 83% of the overall variation in response time. Therefore, findings for the encoding phase do not show any effects for Emotion or Age of faces.

### Memory task (test phase)

#### Reliability analysis

Using 5000 random splits, the Spearman-Brown corrected RT reliability estimates for seen and novel faces were 0.76, 95% CI [0.71, 0.8] and 0.79, 95% CI [0.76, 0.83] respectively. The corrected accuracy reliability estimates for seen and novel faces were 0.22, 95% CI [0.03, 0.39] and 0.33, 95% CI [0.19, 0.43] respectively (see Supplementary Materials, Figs. S1 and S2 for details). Overall, these results indicated a good reliability^46^ for RT and moderate reliability for the Pr measures.

#### Bootstrapping analysis

**Figure 1:**
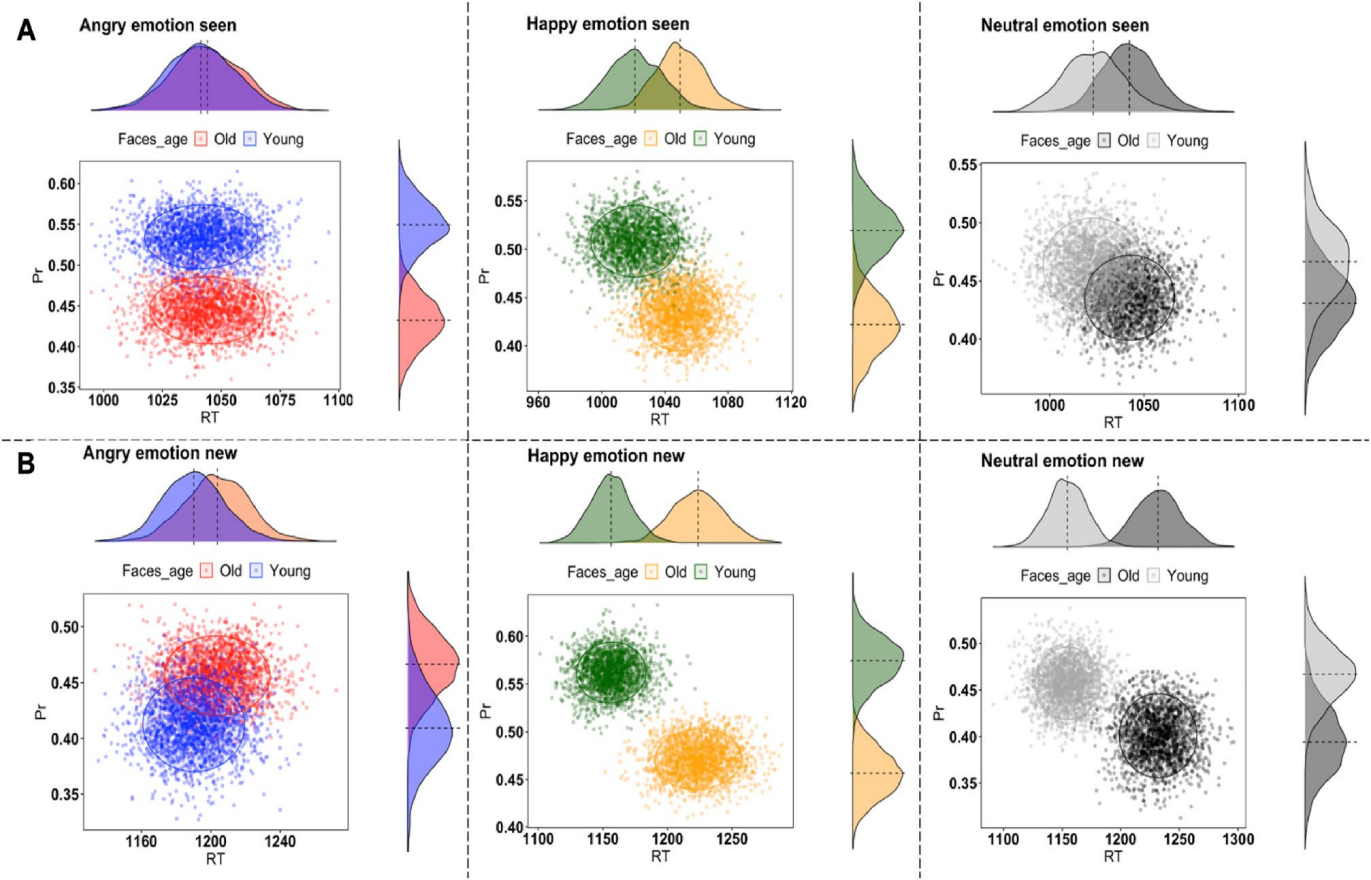
Bootstrapped means of RT (X-axis) and accuracy (Y-axis) for seen (**A**) and novel faces (**B**) in the test phase for Angry (left column), Happy (middle column) and Neutral faces of old and young individuals. Corresponding density plots visualize the distributional overlap of the effects for RT (top plots) and accuracy.

The bootstrapping procedure for Seen faces indicated that RT distribution-clouds largely overlap between old and young faces for emotional (Angry, Happy) and neutral faces (Fig. 1A). This suggests that the Own-Age Bias for faces is unlikely to be driven by differences in speed of responding. In contrast, the distribution-clouds for recognition accuracy (Pr) for emotional faces (Angry, Happy) do not overlap, indicating that participants were more accurate in recognising own-age faces. For Novel faces, RT distribution-clouds for Happy and Neutral faces do not overlap (Fig. 1B). Moreover, the RT distribution-clouds for younger faces are located on the left, suggesting that participants recognised younger faces faster than old ones. The Own-Age Bias for Happy faces is also evident in recognition accuracy (Pr), where the distribution-clouds for young and old faces shows a non-overlapping pattern. In contrast, distribution-clouds for Angry faces are largely overlapping for RT and recognition accuracy (Pr) suggesting no Own-Age Bias.

#### Linear mixed modelling

We tested these effects using four separate LMMs (two on recognition accuracy and two on response time) for seen and novel faces.

#### Performance accuracy (Pr) for seen faces

**Figure 2:**
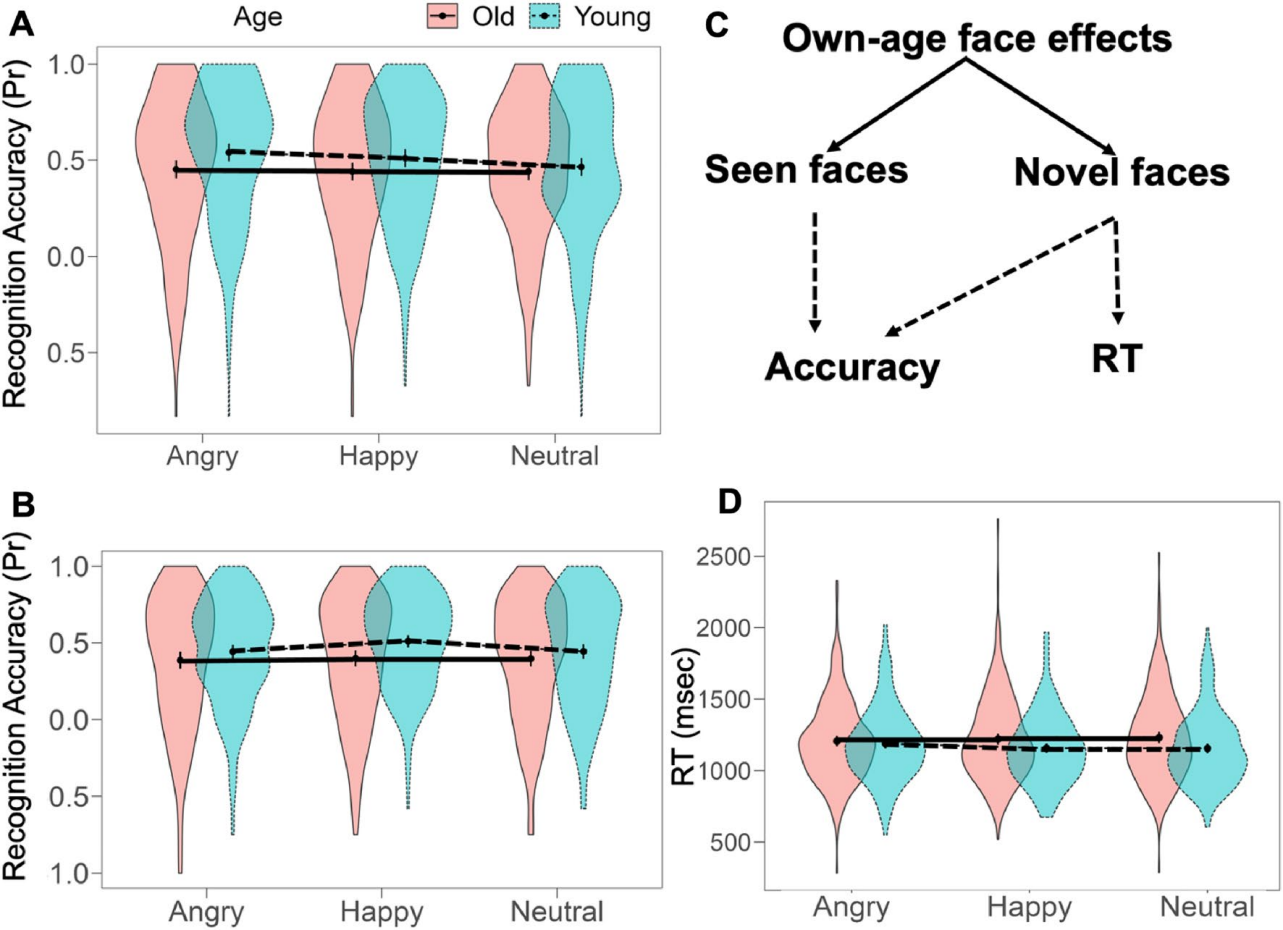
Violin plots depicting the Own Age Bias in recognition accuracy for Seen (**A**) and Novel faces (**B**), and response time for Novel faces (**D**). The black dots represent means of the estimates with 95% confidence intervals. Panel (**C**) represents a schematic summary of the measurements showing the own-age biases in faces.

A LMM on Pr for Seen Faces showed a main effect of Age of faces (F(1,1170)=20.06, *p*<.001): participants were more accurate in recognising younger, seen faces compared to old, seen faces [Young-(Old)], B=0.06, 95% CI [0.03, 0.09), SE=0.01, t(1170)=4.48, p_holm_ <.001). The Emotion term was also significant (F(2,1170)=3.35, *p*=.035) indicating that participants were more accurate in recognising emotional faces compared to neutral faces, [Neutral-(Angry, Happy)], (B=0.03, 95% CI [0.00, 0.06], SE=0.01, t(1170)=2.28, *p*=.023) (Fig. 2A). There were no differences between Angry and Happy faces; [Angry-(Happy)], (B=0.02, 95% CI [-0.01, 0.05], SE=0.02, t(1170)=1.23, *p*=.22). The interaction term (Age by Emotion) was non-significant (F(2,1170)=2.14, *p*=.12). A random effect of Subjects (LRT=537.41, df=1, *p*<.001) contributed 51% to the overall variation in accuracy.

#### Response times (RTs) for seen faces

A LMM on RTs for Seen Faces revealed no significant fixed effect of Age F(1,1170)=3.48, *p*=.06 and Emotion (F(2,1170)=0.47, *p*=.62. The Emotion by Age interaction was also non-significant, F(2)=1.04, *p*=.35. A random effect of Subjects (LRT=604.79, df=1, *p*<.001) explained 54% of the overall variation in response time. Therefore, although for Seen Faces, recognition accuracy is greater for emotional faces of all ages, the typical Own Age Bias with greater recognition accuracy for young faces is present.

#### Performance accuracy (Pr) for novel faces

All three fixed terms in a LMM on Pr for Novel Faces were significant (Age: F(1,1170)=84.91, *p*<.001; Emotion: F(2,1170)=10.45, *p*<.001; Age by Emotion interaction: F(2,1170)=6.86, *p*=.001 (Fig. 2B). The main effect of Age of faces was due to greater recognition accuracy for own-age faces, [Young-(Old)], B=0.07, 95% CI [0.06, 0.09], SE=0.01, t(1170)=9.21, *p*<.001). The main effect of Emotion showed no differences in recognition accuracy between neutral and emotional faces, [Neutral-(Angry, Happy)], B= -0.01, 95% CI [ − 0.03, 0.00], t(1170)=1.70, *p*=.09). However, recognition accuracy was greater for happy compared to angry faces, [Angry-(Happy)], B= − 0.04, 95% CI [− 0.06, − 0.02], SE=0.01, t(1170)= − 4.27, *p*<.001). The interaction term was driven by greater recognition accuracy for younger emotional faces, [Neutral - (Angry, Happy)*Old-Young], B=0.04, 95%CI [0.00, 0.07], SE=0.02, t(1170)=2.26, *p*=.02). Moreover, recognition accuracy was greater for happy, young faces compared to angry, young faces, [Angry-(Happy)*Old-Young], B=0.06, 95% CI [0.02, 0.09], SE=0.02, t(1170)=2.94, *p*=.003). An additional analysis of simple effects of Emotion for old faces showed no effects on recognition accuracy, [Neutral-(Angry, Happy)], B=0.00, 95% CI [− 0.02, 0.03] and [Angry-(Happy)], B= − 0.01, 95% CI [− 0.04, 0.01]). A random effect of Subjects (LRT=1820, df=1, *p*<.001) contributed to 85% of the overall variation in accuracy for novel faces.

#### Response times (RTs) for novel faces

A LMM on RTs revealed no effect of Emotion (F(2,1167)=0.25, *p*=.078). There was an effect of Age of faces (F(1,1167)=27.43, *p*<.001): participant were faster in responding to young faces, [Old-(Young)], B=52.39, 95% CI [32.78, 72.00], SE=10.0, t(1167)=5.24, *p*<.001) (Fig. 2D). A fixed effect omnibus F-test showed a significant Age by Emotion interaction (F(2,1164)=3.14, *p*=.04). However, estimates for two contrasts ([Neutral-(Angry, Neutral)*Old-Young] and [Angry-(Happy)*Old-Young] were not reliable (B=35.34, 95% CI [6.26, − 76.94], SE=21.22, t(1167)=1.67, *p*=.09; B=42.92, 95% CI [− 2.11, 93.95, t(1167)=1.87, *p*=.06).

In sum, findings show that Own-Age Bias is evident for both recognition accuracy and response time (Fig. 2C).

### Loneliness and number of contacts

The mean of loneliness scores across the sample was 24.12 (SD=6.12). Participants reported significantly fewer contacts with young people compared to number of contact with old people (MD=5.5, SE=0.22, 95%CI [5.04, 5.92], t(235)=24.51, *p*<.001, Cohen’s d = 1.59, 95% CI [1.40, 1.79]. Multiple linear regression using backward data entry indicated that only number of contacts with young people could predict loneliness scores (B=0.63, 95%CI [0.23, 1.04], t=3.06, *p*=.002) and the model yielded better fit after removing number of contacts with older people as a predictor (B=0.19, 95%CI [− 0.05, 0.44], t=1.61, *p*=.11) (see Supplementary Materials, Figs. S3 and S4, Tables S1 and S2 for details).

### Loneliness and own-age bias for faces

A multiple regression model showed that Own-Age Biases in recognition accuracy for Seen Angry (B=1.47, SE=1.17, 95% CI [− 0.84, 3.78], t=1.25, *p*=.21), Novel Angry (B=0.24, SE=1.21, 95%CI [− 2.14, 262], t=0.19, *p*=.85), and Seen Happy faces (B=− 1.99, SE=1.19, 95% CI [− 4.33, 0.36], t=− 1.67, *p*=.09) were not reliable predictors of loneliness. There was a small effect of Own-Age Biases in recognition accuracy for Novel Happy faces on Loneliness scores (B=− 4.3844, SE=2.074, 95%CI [− 8.46, − 0.29], t=2.1, *p*=.036) indicating that stronger Own-Age Biases are associated with less loneliness (see Supplementary Materials, Fig. S5, Table S3 for details).

## Discussion

We investigated whether the Own Age Bias—that is the better recognition for faces of individuals of one’s own age—that has been consistently observed with neutral faces, also occurs with emotional faces. In addition, we assessed to what extent self-reported loneliness and number of social contacts modulate the Own Age Bias for neutral and emotional faces. To this aim, young individuals completed a two-phase experiment, in which they categorised faces of old and young individuals showing neutral, happy, and angry expressions based on age (young vs. old). In the test phase, participants were presented with already seen and novel faces. Their task was to assess for each face whether they had previously seen it.

Findings for the whole sample show an Own Age Bias for both seen faces (i.e., greater accuracy for young faces) and for novel faces (i.e., greater accuracy and faster responses for young faces). Importantly, although recognition accuracy was better for emotional faces, the Own Age Bias occurred for neutral as well as for emotional faces. In addition, when looking at whether loneliness affects the Own Age Bias, findings showed that individuals who reported less loneliness were more likely to show an Own Age Bias for novel happy faces, expressing social affiliation. Although the effect is small, this is an interesting finding considering that induced social exclusion (via the Cyberball task) has been linked to increased attention to signals of possible social reconnection^24^ and loneliness has been linked to increased attention toward signals of social threat^28^. Therefore, the implications of the present findings are twofold. Firstly, we observed an Own Age Bias with neutral and emotional faces, whereas Ebner and Johnson^18^ and Cronin et al.^19^ did not. We believe that this could be due to differences in the task used at encoding. In fact, although in their meta-analysis Rhodes and Anastasi^13^ concluded that task requirements were not predictive of the magnitude of the Own Age Bias, this conclusion may well apply to studies using neutral faces only. This is because with neutral faces there is only one salient dimension (i.e., age) whereas with emotional faces of young and old individuals there are two salient dimensions (i.e., age and emotion). We used an age categorization task at encoding based on evidence^8^ that this task, by emphasizing the salient age-dimension, enhances the own-age memory bias. In contrast, Ebner and Johnson^18^ used an emotion categorization task and Cronin et al.^19^ used a passive viewing task and the instructions to memorize the face and press a key as soon as the face disappeared from screen. This would point to the importance—when assessing the Own Age Bias—of using task instructions that emphasise the salience of the age dimension especially when there is a competing salient dimension (i.e., emotion). However, our study differed from past studies also about the stimuli used and how performance was assessed. In fact, our stimuli were balanced for distinctiveness as this can affect memory performance^47^ and we opted for assessing performance by using the discrimination index (i.e., Pr) separately for seen and novel faces. This is as in Denkinger and Kinn^17^, whereas Ebner and Johnson^18^ and Cronin et al.^19^ used d’ as a combined-overall performance for seen and new faces. However, it has been pointed out that d’ can produce extremely biased predictions for recognition performance^48^.

Given that we observed an Own Age Bias for neutral as well as for emotional faces, the second research question is whether loneliness affects the Own Age Bias. Our findings show that for young individuals, it is the number of social contacts with their peers that is associated with loneliness and that lower loneliness is characterized by a larger Own Age Bias for novel signals of social affiliation (i.e., novel happy faces), which speaks in favour of contact-based accounts (i.e., the perceptual-expertise account) of the Own Age Bias. The other side of the coin being that this bias is not present with higher levels of loneliness, suggesting that what loneliness does is to reduce the natural tendency toward new signals of social connections from one’s peers, which may contribute to maintaining the causes of loneliness. This is an important and novel finding considering that evidence indicates a bias toward signals of social connection in response to momentary social exclusion^24,25^ but that loneliness is linked to a bias is toward social threat signals^26–28^. Although past findings refer to attentional biases whereas the present study is the first to assess the effect of loneliness on the Own Age Bias, it is quite possible that loneliness affects a wide range of social cognition processes. In fact, recent evidence shows that high levels of loneliness in young individuals is associated with changes in brain areas (i.e., stronger functional connectivity between the inferior frontal gyrus and the supplementary motor area, the precentral gyrus, the superior parietal lobule) involved in social attention^49^.

To conclude, the present findings indicate that the Own Age Bias occurs for neutral as well as emotional faces in young individuals, provided the age of faces is salient for the task at hand. In addition, for young individuals the number of social contacts with their age peers is important as it predicts loneliness. Finally, only young individuals with less loneliness show an Own Age Bias for novel, happy faces. This points to a typical bias toward new signals of social affiliation that is lacking or reduced in individuals with more self-reported loneliness.

Despite the strengths of the present study, we should also acknowledge some limitations, that may be addressed by future research. Our participants reported moderate levels of loneliness and future research should address whether our findings generalize to high levels of loneliness. Alternatively, higher loneliness could be linked to an Own Age Bias for social threat signals. In addition, our study was conducted using an online platform (as was the study by Cronin et al.^19^, during the lockdown due to Covid-19 pandemic. Albeit we used video-calls via Google Meet to ensure that participants completed the task under relatively controlled conditions (i.e., being alone in the room, being in quiet environment, etc.) a certain variability between conditions could still be present. In addition, loneliness was assessed with the UCLA Loneliness Scale due to its validity and reliability. However, this questionnaire assesses overall loneliness whereas loneliness may have multiple subcomponents. Moreover, self-report measures may be prone to social desirability^50^ although, using standardized questionnaires allows to counteract these issues. Importantly, that the study was conducted during the lockdown due to the Covid-19 pandemic (the study took place during semester 2, 2021 when lectures were all in remote-modality) and that participants completed the study remotely, may have made it more socially acceptable for students to report their feelings of loneliness as they could be attributed to the objective and extreme conditions. Indeed, for a separate study in our laboratory we have recently experienced that it is particularly challenging to motivate individuals with high levels of loneliness to take part in laboratory-based studies as we tried to select participants based on their results to the questionnaire completed online, but we had to change our strategy as there was a very low response-rate.

Understanding the effects of loneliness on different aspects of social cognition is important, but it has become more urgent recently as especially young individuals have suffered from the lack of social contacts with their peers during the lockdown due to Covid-19 pandemic. Future research on the effects of loneliness on social cognition could help understanding whether the mechanisms that contribute to maintaining loneliness are characterized first by reduced bias toward novel social affiliation signals, followed by hypervigilance for novel social threat signals.

## Supplementary Information

Supplementary Information.

Supplementary Information.

## Data Availability

All data generated or analysed during this study are included in this published article and its supplementary information files.
