# Supplementary material for: Social contacts and loneliness affect the own age bias for emotional faces
Source: Sci Rep. 2022 Sep 27;12:16134. doi: 10.1038/s41598-022-20220-9 (PMC9514703; doi:10.1038/s41598-022-20220-9)
Supplement: Supplementary file 1 — Supplementary Information. [file 41598_2022_20220_MOESM1_ESM.docx]

**Supplementary Materials**

1. List of Faces used
2. Reliability analyses: see Supplementary Materials, Fig. S1 and Fig. S2 for details).
3. Loneliness and number of contacts: see Supplementary Materials for details
4. Multiple regression model showed that Own-Age Biases in recognition accuracy: see Supplementary Materials for details
