# Supplementary material for: Social contacts and loneliness affect the own age bias for emotional faces
Source: Sci Rep. 2022 Sep 27;12:16134. doi: 10.1038/s41598-022-20220-9 (PMC9514703; doi:10.1038/s41598-022-20220-9)
Supplement: Supplementary file 2 — Supplementary Information. [file 41598_2022_20220_MOESM2_ESM.docx]

**Supplementary Materials**

List of stimuli (face identities) selected from FACES dataset:

Young adult females: 010, 022, 034, 071, 085, 090, 101, 125, 132, 150, 162, 177

Old adult females: 012, 021, 030, 036, 050, 060, 067, 096, 112, 124, 158, 164

Young adult males: 037, 049, 066, 081, 089, 105, 114, 123, 135, 167, 170

Old adult males: 004, 015, 027, 033, 042, 083, 102, 118, 141, 146, 166, 176

Reliability analysis

Using 5000 random splits, the Spearman-Brown corrected reliability estimates for seen and novel faces were 0.76, 95% CI [0.71, 0.8] and 0.79, 95% CI [0.76, 0.83] respectively (Fig. S1).


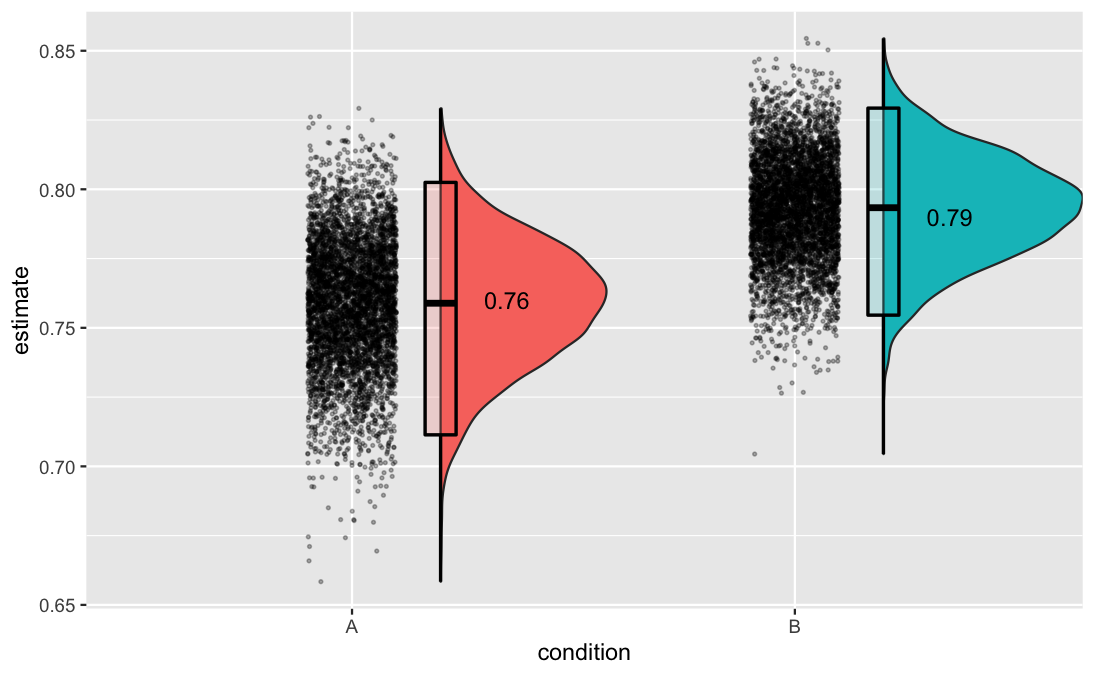


Figure S1. Internal reliability estimates in Test phase for response time. Condition A represent seen faces, condition B represents novel faces. Graphs depict estimate density plots with mean and 95% confidence interval.

The corrected recognition scores, reliability estimates for seen and novel faces were 0.22, 95% CI [0.03, 0.39] and 0.33, 95% CI [0.19, 0.43] respectively (s Fig.S2).


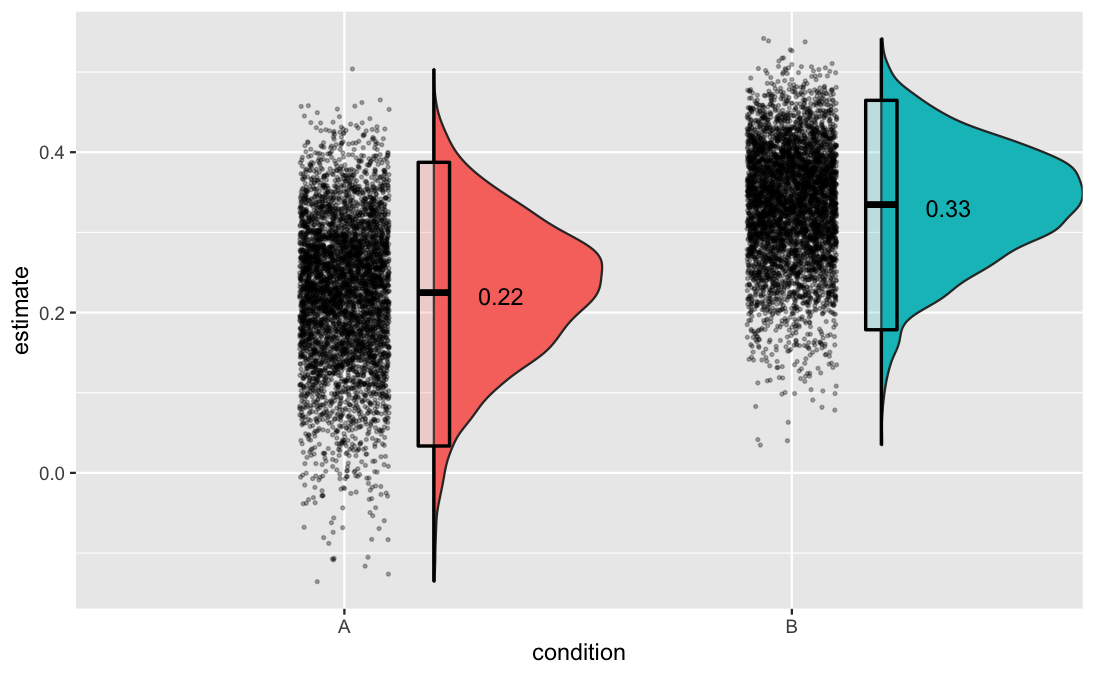


Figure S2. Internal reliability estimates in Test phase for corrected recognition scores. Condition A represent seen faces, condition B represents novel faces. Graphs depict estimate density plots with mean and 95% confidence interval.

Associations between the loneliness scores (UCLA) and number of contacts with young and old people


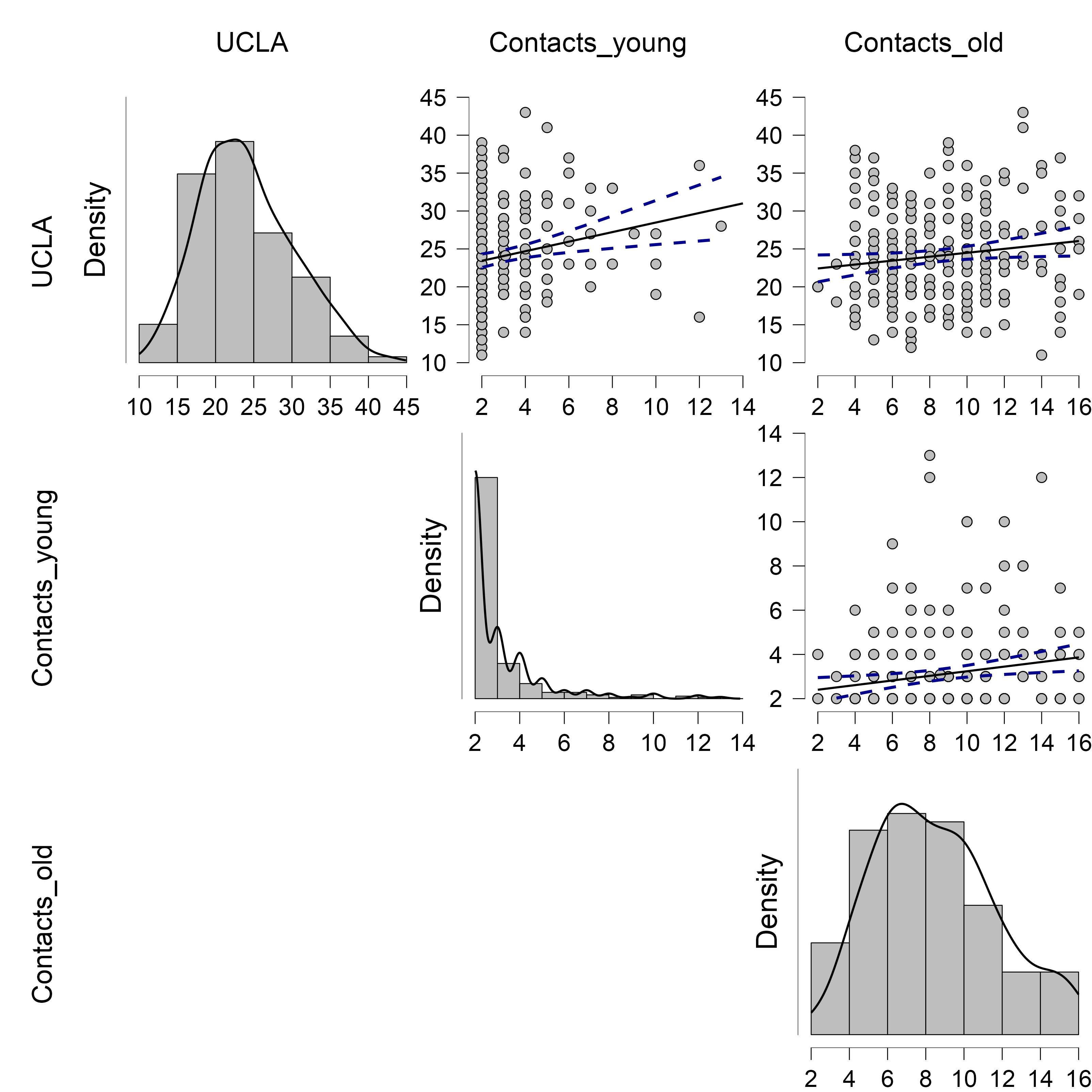


Figure S3. Scatter plots depicting associations between the loneliness scores (UCLA) and number of contacts with young and old people. The X-axis represents number of contacts, Y=axis represents UCLA scores.


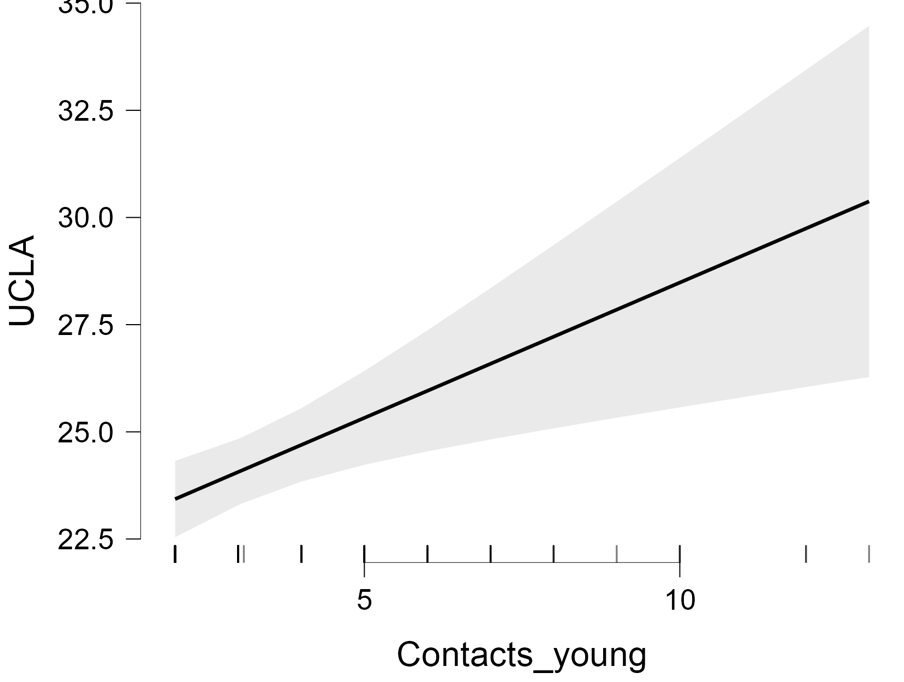


Figure S4. Marginal effect of Contacts with young people on UCLA scores

| Table S1. ANOVA | | | | | | | | | | | | | |
| --- | --- | --- | --- | --- | --- | --- | --- | --- | --- | --- | --- | --- | --- |
| Model | |  | | Sum of Squares | | df | | Mean Square | | F | | p | |
| 1 |  | Regression |  | 432.596 |  | 2 |  | 216.298 |  | 6.013 |  | 0.003 |  |
|  |  | Residual |  | 8382.067 |  | 233 |  | 35.975 |  |  |  |  |  |
|  |  | Total |  | 8814.664 |  | 235 |  |  |  |  |  |  |  |
| 2 |  | Regression |  | 339.904 |  | 1 |  | 339.904 |  | 9.385 |  | 0.002 |  |
|  |  | Residual |  | 8474.760 |  | 234 |  | 36.217 |  |  |  |  |  |
|  |  | Total |  | 8814.664 |  | 235 |  |  |  |  |  |  |  |
|  | | | | | | | | | | | | | |

| Table S2. Coefficients | | | | | | | | | | | | | | | | | | | | | |
| --- | --- | --- | --- | --- | --- | --- | --- | --- | --- | --- | --- | --- | --- | --- | --- | --- | --- | --- | --- | --- | --- |
|  | | | | | | | | | | | | | | 95% CI | | | | Collinearity Statistics | | | |
| Model | |  | | Unstandardized | | Standard Error | | Standardized | | t | | p | | Lower | | Upper | | Tolerance | | VIF | |
| 1 |  | (Intercept) |  | 20.649 |  | 1.205 |  |  |  | 17.137 |  | < .001 |  | 18.275 |  | 23.023 |  |  |  |  |  |
|  |  | Contacts_young |  | 0.572 |  | 0.209 |  | 0.178 |  | 2.743 |  | 0.007 |  | 0.161 |  | 0.983 |  | 0.969 |  | 1.032 |  |
|  |  | Contacts_old |  | 0.199 |  | 0.124 |  | 0.104 |  | 1.605 |  | 0.110 |  | -0.045 |  | 0.443 |  | 0.969 |  | 1.032 |  |
| 2 |  | (Intercept) |  | 22.169 |  | 0.747 |  |  |  | 29.661 |  | < .001 |  | 20.697 |  | 23.642 |  |  |  |  |  |
|  |  | Contacts_young |  | 0.631 |  | 0.206 |  | 0.196 |  | 3.064 |  | 0.002 |  | 0.225 |  | 1.037 |  | 1.000 |  | 1.000 |  |
|  | | | | | | | | | | | | | | | | | | | | | |

Associations between the loneliness scores (UCLA) and own-age biases in recognition scores

| Table S3. Model Coefficients - UCLA | | | | | | | | | | | | | | | | | | | |
| --- | --- | --- | --- | --- | --- | --- | --- | --- | --- | --- | --- | --- | --- | --- | --- | --- | --- | --- | --- |
|  | | | | | | 95% Confidence Interval | | | |  | | | | | | 95% Confidence Interval | | | |
| Predictor | | Estimate | | SE | | Lower | | Upper | | t | | p | | Stand. Estimate | | Lower | | Upper | |
| Intercept |  | 24.62 |  | 0.48 |  | 23.67 |  | 25.58 |  | 50.86 |  | < .001 |  |  |  |  |  |  |  |
| Novel_Happy_bias |  | -4.44 |  | 2.04 |  | -8.46 |  | -0.43 |  | -2.18 |  | 0.030 |  | -0.15 |  | -0.28 |  | -0.01 |  |
| Seen_Happy_Bias |  | -1.92 |  | 1.13 |  | -4.15 |  | 0.32 |  | -1.69 |  | 0.092 |  | -0.12 |  | -0.26 |  | 0.02 |  |
| Seen_Angry_Bias |  | 1.53 |  | 1.13 |  | -0.71 |  | 3.76 |  | 1.35 |  | 0.180 |  | 0.10 |  | -0.04 |  | 0.24 |  |
|  | | | | | | | | | | | | | | | | | | | |


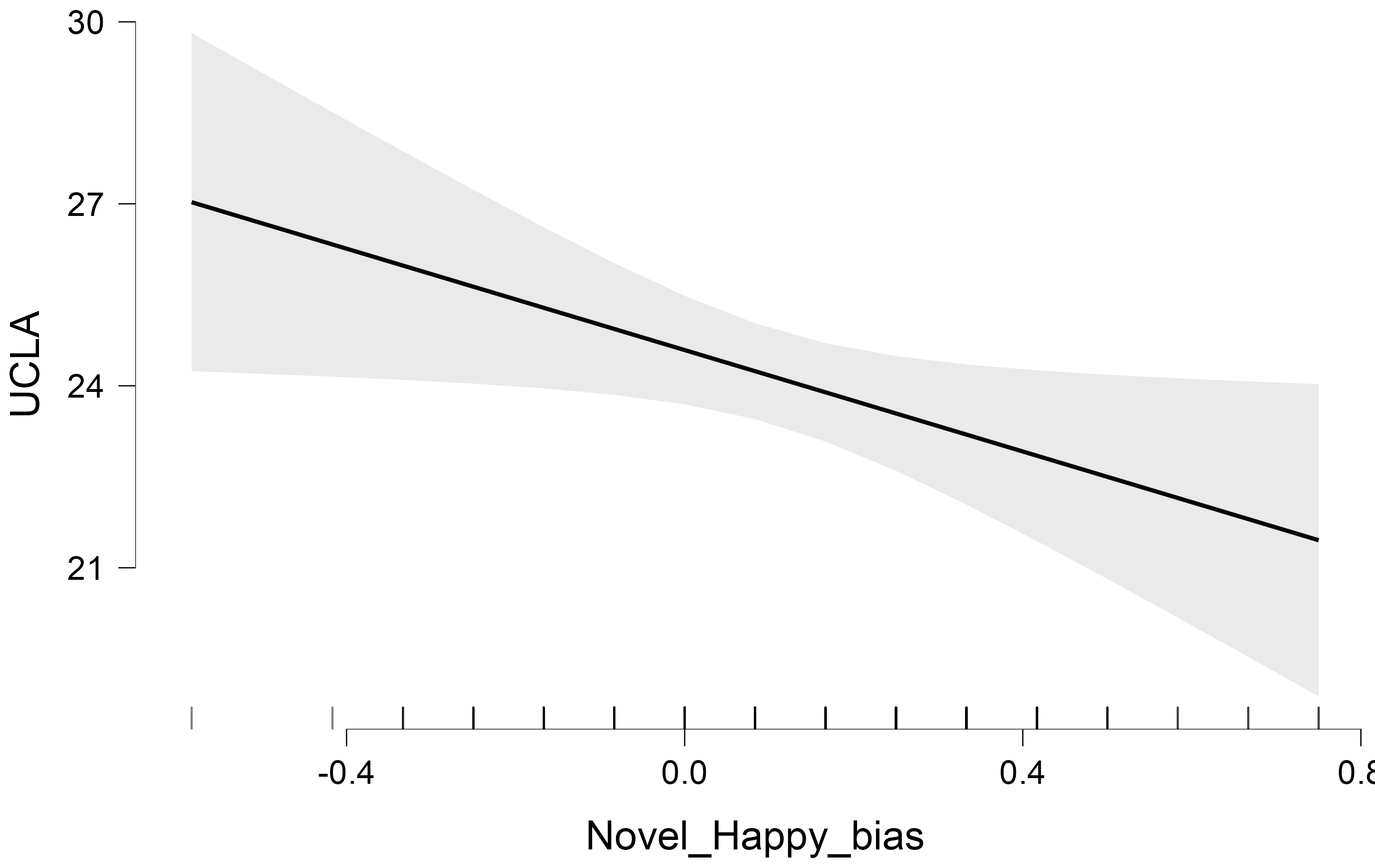


Figure S5. Marginal effect of Novel Happy bias on UCLA
